# Association of Vascular Age and Subclinical Target Organ Damage in a Beijing Community-Based Population: A Cross-Sectional Study

**DOI:** 10.3390/jcdd13010056

**Published:** 2026-01-21

**Authors:** Xiangning Zhang, Lan Gao, Fangfang Fan, Jia Jia, Tianhui Dong, Yang Yu, Yan Zhang

**Affiliations:** 1 Department of Cardiology, Peking University First Hospital, Beijing 100034, China; 2 Institute of Cardiovascular Disease, Peking University First Hospital, Beijing 100034, China

**Keywords:** vascular aging, arterial stiffness, target-organ damage

## Abstract

Background: Vascular aging (VA) reflects arterial biological aging and is closely linked to cardiovascular risk. Carotid–femoral pulse wave velocity (cfPWV) is the gold standard for assessing arterial stiffness and VA. However, evidence is limited on cfPWV-derived vascular age and its association with subclinical target organ damage (TOD) in the general population. This study evaluated whether Δ-age (vascular age minus chronological age) could identify individuals at higher risk of early vascular injury in a Chinese community cohort. Methods: This cross-sectional study included participants from two Beijing communities. Δ-age was calculated as cfPWV-derived vascular age minus chronological age. Participants were categorized as supernormal vascular aging (SUPERNOVA, <10th percentile), normal VA, and early vascular aging (EVA, 90th percentile). TOD included mean and maximum carotid intima-media thickness (CIMT), and carotid plaque. Associations between Δ-age and TOD were analyzed using multivariable regression models adjusted for conventional cardiovascular risk factors and cfPWV. Results: A total of 6305 participants (mean age 62.5 ± 7.8 years; 34.2% male) were included. Higher Δ-age was associated with increased mean and maximum CIMT and higher carotid plaque prevalence, independent of cfPWV. EVA participants had a higher risk, whereas SUPERNOVA participants had a lower risk of TOD compared with normal VA. After cfPWV adjustment, EVA remained associated with increased mean CIMT and carotid plaque, while SUPERNOVA showed a nonsignificant trend toward a lower risk. Associations were consistent across subgroups. Conclusions: Δ-age, independent of cfPWV, was an independent risk factor for TOD. This simple, practical indicator may help identify individuals at risk of early vascular damage in community settings.

## 1. Introduction

VA represents the manifestation of biological aging at the vascular level. Although this process progresses naturally with increasing chronological age, previous longitudinal cohort studies have shown that age-related alterations in vascular properties can be detected across nearly the entire lifespan [1,2,3,4]. Moreover, VA can be accelerated by various risk factors, including elevated systolic blood pressure, increased body mass index, hypercholesterolemia, smoking, and higher resting heart rate, leading to substantial heterogeneity inVA among individuals [5,6,7]. Evidence from prior studies further indicates that accelerated VA is strongly associated with cardiovascular events [8,9,10]. Collectively, these findings suggest that vascular age may serve as an early indicator for identifying individuals at higher cardiovascular risk.

Previous studies investigating the relationship between cfPWV-derived vascular age and subclinical organ damage have been limited. Ji et al. conducted a study among community-dwelling elderly individuals in China, demonstrating that increased cfPWV, estimated from participants’ age and blood pressure, was associated with preclinical left ventricular diastolic dysfunction and microalbuminuria [11]. However, cfPWV is strongly age-dependent, making it difficult to distinguish physiological VA from pathological acceleration when absolute cfPWV values are interpreted alone. By transforming cfPWV into an age-equivalent metric, vascular age contextualizes arterial stiffness relative to chronological age and enables the quantification of inter-individual deviations in VA.

There may exist a complex interplay among VA, TOD, and the development of cardiovascular disease (CVD). A cross-sectional study by Vasan et al. demonstrated that elevated arterial stiffness, assessed by cfPWV, contributes to TOD and partially mediates its relationship with incident CVD [12]. These findings underscore the importance of early detection of arterial stiffness in preventing organ damage and halting the transition from subclinical abnormalities to overt cardiovascular disease. Building upon this concept, our study further incorporated the notion of vascular age, taking into account the natural aging process of the vasculature. In this context, Δ-age, defined as the difference between vascular age and chronological age, reflects the extent to which an individual’s vascular condition deviates from the expected age-related norm, rather than the absolute level of arterial stiffness.

Accordingly, this study aimed to investigate the association between cfPWV-derived vascular age and Δ-age with subclinical TOD in a community-based population. We sought to determine whether Δ-age provides incremental information beyond cfPWV in identifying individuals with early vascular-related organ damage.

## 2. Materials and Methods

### 2.1. Study Population

Participants in this cross-sectional study were derived from an atherosclerosis cohort survey conducted in the Gucheng and Pingguoyuan communities of the Shijingshan District, Beijing, China, between December 2011 and April 2012. The cohort design and recruitment procedures have been described previously [13]. A total of 6568 individuals who participated in the 7th-year on-site follow-up from September to December 2018 were considered eligible for inclusion. Among them, 263 were excluded due to missing data on vascular age, carotid plaque, and quantitative carotid ultrasonography at baseline. Ultimately, 6305 participants were included in the present analysis. The study protocol was approved by the Ethics Committee of Peking University First Hospital, and written informed consent was obtained from all participants.

### 2.2. Clinical Data Collection

A standardized questionnaire was administered to collect demographic data (e.g., sex and age), lifestyle behaviors (e.g., smoking, drinking), medical history (hypertension, coronary heart disease, and stroke, including cerebral infarction or hemorrhage), and medication use (antihypertensive, hypoglycemic, and lipid-lowering drugs). Current smoking was defined as smoking ≥1 cigarette per day for ≥6 months, and current alcohol consumption as drinking ≥1 time per week for ≥6 months.

### 2.3. Physical Examination

Anthropometric measurements included body mass index (BMI), calculated as weight (kg) divided by height squared (m^2^). Blood pressure and pulse rate were measured after 5 min of seated rest using an Omron HEM-7130 electronic sphygmomanometer (Omron Healthcare Co. Ltd, Kyoto, Japan) with appropriately sized cuffs and standard calibration. Three consecutive measurements were taken at ≥1-min intervals, and the averages of SBP, DBP, and pulse rate were recorded [13].

### 2.4. Laboratory Examination

Venous blood samples were collected after an overnight fast of at least 12 h. Serum and plasma were separated within 30 min and stored at −80 °C for measurement of fasting blood glucose (FBG), TC, TG, LDL-C, and HDL-C. Urine samples (15 mL) were collected from the first morning void on the survey day. Urinary microalbumin was measured using the rate-scattering turbidimetric method (Immang 800, Beckman Coulter, Inc., Brea, CA, USA), and urinary creatinine was measured using the picric acid method (AU5800, Beckman Coulter, Inc., Brea, CA, USA) [13].

### 2.5. Carotid Ultrasonography

High-resolution B-mode ultrasound imaging (Terason EchoTM ultrasound system, Terason/Teratech Corporation, Burlington, MA, USA) of bilateral carotid arteries was performed by certified sonographers. Participants were examined in a supine position with the head turned contralaterally at approximately 45°. CIMT was defined as the distance between the lumen-intima and media–adventitia interfaces of the common carotid artery and measured over a 1 cm segment of the far wall at end-diastole. Mean and maximum CIMT values were obtained as the average of bilateral measurements. Carotid plaque was defined as a focal structure encroaching into the arterial lumen by at least 0.5 mm, or 50% greater than the surrounding intima-media thickness, or demonstrating a thickness >1.5 mm from the intima-lumen to the media-adventitia interface. To ensure measurement reliability, CIMT and plaque assessments were performed by operators blinded to participants’ cfPWV and clinical information.

### 2.6. Vascular Age

Vascular age was measured using the PulsePen device (DiaTecne s.r.l., Milan, Italy; www.pulsepen.com), which consists of a high-fidelity tonometer for recording carotid and femoral pulse waveforms and an integrated electrocardiogram (ECG) unit. Trained operators acquired cfPWV following a standardized protocol. Vascular age was automatically calculated by the PulsePen software (version 2.3.1) based on the relationship between cfPWV, chronological age, and the established reference curve for the general population. Δ-age was defined as vascular age minus chronological age. The 10th and 90th percentiles of Δ-age corresponded to −4.50 and +3.50 years, respectively, and were used as cutoffs to define SUPERNOVA and EVA, respectively, while values between these percentiles were considered normal VA [14].

### 2.7. Statistical Analysis

In the description of population characteristics, participants were categorized into three groups: EVA, normal VA, and SUPERNOVA. Continuous variables were expressed as mean ± standard deviation for normally distributed data or as median (interquartile range [IQR]) for skewed data, whereas categorical variables were presented as counts (percentages). Between-group differences were assessed using analysis of variance (ANOVA) or the Kruskal–Wallis rank-sum test for continuous variables, as appropriate. The Pearson χ^2^ test was used for categorical variables, and Fisher’s exact test was applied for those with a theoretical frequency < 10.

Collinearity among covariates was evaluated using the variance inflation factor (VIF), with VIF > 10 indicating problematic multicollinearity. Restricted cubic spline models and multivariable logistic regression analyses were used to assess the cross-sectional associations between Δ-age and indicators of TOD. The regression models were adjusted for age, sex, body mass index, estimated glomerular filtration rate, smoking and drinking habits, use of antihypertensive, hypoglycemic, and lipid-lowering drugs, and history of hypertension, diabetes, hyperlipidemia, myocardial infarction, and stroke. To evaluate whether the observed associations were independent of arterial stiffness, an additional model was further adjusted for cfPWV. Subgroup and interaction analyses were also performed by stratifying participants according to traditional cardiovascular risk factors, with β coefficients or odds ratios calculated for each stratum. Sensitivity analyses were performed to assess the robustness of the findings. Specifically, Δ-age was alternatively modeled as a binary variable using 0 as the cutoff (Δ-age ≥ 0 vs. <0) and as quartiles. Multivariable regression models were repeated using these alternative classifications, adjusting for the same covariates as in the primary analyses. All statistical analyses were conducted using Empower (R) (5.2, www.empowerstats.com; X&Y Solutions, Inc., Boston, MA, USA) and R software (version 3.4.3; http://www.R-project.org). A two-sided *p* value < 0.05 was considered statistically significant.

## 3. Results

### 3.1. Baseline Characteristics

Table 1 shows participant characteristics across VA categories: EVA, normal VA, and SUPERNOVA. Participants with EVA and SUPERNOVA were both older than those with normal VA; EVA individuals were younger than SUPERNOVA. Descriptive data of Δ-age and VA categories across 10-year age groups are shown in Supplementary Table S1. Compared with normal VA and SUPERNOVA, EVA participants were more likely to be male, had higher BMI, blood pressure, and fasting blood glucose and were more likely to be current smokers or drinkers and to have a history of hypertension, diabetes, and cardiovascular or cerebrovascular disease. EVA participants exhibited higher cfPWV, carotid plaque prevalence, and CIMT, whereas SUPERNOVA participants showed lower cfPWV.

### 3.2. Associations Between VA Categories and TOD

**Figure 1:**
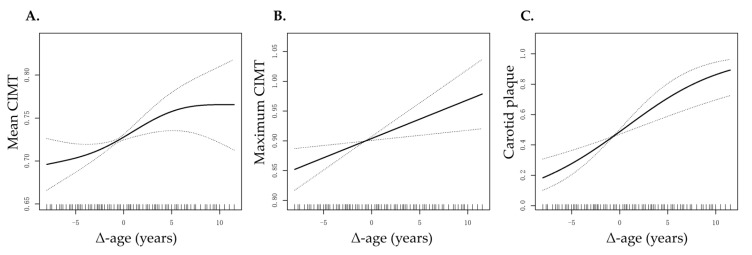
The relationship between Δ-age and target organ damage. Adjusted for age, sex, body mass index, estimated glomerular filtration rate, smoking and drinking habit, use of antihypertensive drugs, hypoglycemic drugs, and lipid-lowering drugs, history of hypertension, diabetes and hyperlipidemia, and carotid-femoral pulse wave velocity. A 2-tailed 0.5% of Δ-age (below −8.50 or above 11.58) was removed. (**A**) Δ-age vs. mean CIMT. (**B**) Δ-age vs. maximum CIMT. (**C**) Δ-age vs. presence of carotid plaque. Abbreviations: CIMT, carotid intima-media thickness.

As illustrated in Figure 1, mean CIMT, maximum CIMT, and prevalence of carotid plaque all increased progressively with higher Δ-age (defined as vascular age minus chronological age, in years) after adjustment for potential confounders, including cfPWV.

In the fully adjusted regression model, collinearity was low (VIF ≤ 3.1). Table 2 presents the multivariable regression results: each 1-unit increase in Δ-age was associated with a 0.003 mm (95% CI: 0.002–0.004) increase in mean CIMT, a 0.004 mm (95% CI: 0.003–0.005) increase in maximum CIMT, and a 6.5% increase in the odds of carotid plaque (OR = 1.065, 95% CI: 1.044–1.085). After further adjustment for cfPWV, the associations remained significant (0.006 mm mean CIMT, 0.008 mm max CIMT, 18.5% higher odds of plaque).

When Δ-age was categorized, participants with EVA showed significantly higher mean and maximum CIMT (β = 0.030, 95% CI: 0.020–0.040; β = 0.031, 95% CI: 0.019–0.044, respectively) and greater odds of carotid plaque (OR = 1.752, 95% CI: 1.418–2.165) compared with those with normal VA. In contrast, participants with SUPERNOVA had lower mean and maximum CIMT (β = −0.010, 95% CI: −0.019 to −0.000; β = −0.016, 95% CI: −0.027 to −0.004, respectively) and reduced odds of carotid plaque (OR = 0.815, 95% CI: 0.668–0.994). After cfPWV adjustment, EVA remained associated with mean CIMT (β = 0.016) and plaque (OR = 1.419), with consistent trends observed for maximum CIMT and SUPERNOVA participants (Table 2).

Associations remained consistent after adjustment for UACR and combined CKD G-A staging; sensitivity analyses (binary and quartile Δ-age) and stratified subgroups showed consistent directionality without significant interactions; details are provided in the Supplementary (Tables S2–S6).

## 4. Discussion

In our study, Δ-age, defined as the difference between cfPWV-derived vascular age and chronological age, was significantly associated with carotid arterial damage, with consistent associations across subgroups stratified by traditional cardiovascular risk factors.

A variety of methods have been developed to assess vascular age, including imaging, circulating biomarkers, metabolic indicators, and pulse wave velocity (PWV) [15]. Although no single measure has been universally accepted as the gold standard, PWV remains a classical marker of arterial stiffness with well-established predictive value for cardiovascular events [16,17,18,19]. Among PWV measurement approaches, cfPWV is recognized as the gold standard for noninvasive assessment and is endorsed by clinical guidelines [20,21,22,23]. Evidence also links cfPWV with cardiovascular disease incidence [24,25,26], supporting its use to quantify VA. Therefore, integrating cfPWV into the concept of vascular age allows arterial stiffness to be interpreted relative to an individual’s chronological age, thereby identifying individuals whose VA exceeds the expected physiological trajectory.

Previous studies have shown associations between cfPWV and subclinical organ damage, particularly in both hypertensive and general populations [27,28]. Building upon this evidence, our study combined cfPWV with vascular age to identify individuals at higher risk of vascular injury in the general population.

From a practical perspective, Δ-age may represent a clinically intuitive and scalable tool for vascular risk screening. Because cfPWV measurement is already used in research and increasingly available in clinical settings, Δ-age can be readily derived without additional testing. Unlike fixed PWV thresholds, Δ-age contextualizes arterial stiffness within an age-adjusted framework, which may improve identification of high-risk individuals. This approach may be especially useful in population-based screening, although its incremental predictive value over established risk scores requires confirmation in prospective studies.

In our present study, we further adjusted for cfPWV in the multivariable analysis of the association between Δ-age and TOD, demonstrating that the effect of Δ-age is independent of cfPWV—a finding not previously reported in the literature. This suggests that cfPWV-derived vascular age reflects aspects of vascular aging not fully captured by cfPWV alone.

Carotid plaque was included as a key marker of subclinical atherosclerosis; however, plaque presence alone does not fully capture cardiovascular risk. Increasing evidence indicates that plaque vulnerability—such as lipid-rich necrotic cores, thin fibrous caps, and intraplaque neovascularization—may also be strongly associated with adverse cardiovascular outcomes than plaque burden per se [29,30]. Age-related changes in plaque composition may further modify this risk, potentially linking accelerated VA to plaque instability rather than mere plaque formation [31]. Future studies incorporating plaque morphology or vulnerability indices may provide deeper insight into the relationship between VA and atherosclerotic risk.

Regarding collections of other cofounders, therapeutic interventions may influence vascular parameters and plaque burden. Statin therapy has been shown not only to reduce atherosclerotic plaque volume but also to favorably remodel plaque composition, including thickening of the fibrous cap and reducing lipid-rich necrotic core, thereby lowering plaque vulnerability, effects that may not be fully captured by CIMT alone [32,33,34]. Given the heterogeneity in statin exposure in real-world settings—particularly with respect to treatment intensity, initiating age, and duration—such therapy-related effects may modify cross-sectional associations between Δ-age and plaque presence [35]. Moreover, antihypertensive and glucose-lowering therapies may also affect arterial stiffness and endothelial function [36,37]. Although medication use was adjusted for in our analyses, detailed information on treatment duration and intensity was unavailable, which may have resulted in residual confounding.

Increasing studies have indicated that oxidative stress represents another important mechanism linking VA by promoting endothelial dysfunction, inflammation, and smooth muscle cell proliferation, thereby accelerating both arterial stiffening and atherosclerotic progression [38]. Although oxidative stress markers were not measured in this study, their contribution may partially underlie the observed associations between Δ-age and TOD.

Several limitations warrant consideration. First, the cross-sectional design precludes causal inference; longitudinal studies are needed to determine whether Δ-age predicts progression of TOD and future cardiovascular events. Second, this study was conducted in a single community-based cohort in Beijing, which may limit generalizability. Third, the study population consisted predominantly of middle-aged and older adults, and validation in younger populations is needed. Fourth, while TOD generally involves multiple organ systems, this study focused mainly on carotid artery–based vascular markers. Although these measures are well-established indicators of subclinical vascular injury, they may not comprehensively reflect damage in other target organs. Future longitudinal studies with integrated multi-organ phenotyping may provide a more complete understanding of the relationship between VA and systemic TOD. Fifth, this study lacked detailed information on the duration and intensity of antihypertensive, glucose-lowering, and lipid-lowering therapies; therefore, future studies of Δ-age should standardize treatment exposure—such as statin intensity class, time on therapy, and cumulative dose or defined daily dose—to better disentangle pharmacologically driven vascular remodeling from true acceleration of vascular aging. Finally, future studies incorporating longitudinal follow-up, detailed treatment exposure, plaque vulnerability assessment, and mechanistic biomarkers such as oxidative stress may further clarify the clinical utility of Δ-age as a marker of VA.

## 5. Conclusions

Our findings show that Δ-age, calculated as vascular age minus chronological age, was independently associated with markers of early vascular damage in a Chinese community-based population, with consistent associations across the different cardiovascular risk factor stratifications. Δ-age derived from cfPWV distribution in the general population may reflect relative deviations in vascular aging and could provide complementary information, although its potential role in cardiovascular risk assessment and early identification requires confirmation in longitudinal studies.

## Figures and Tables

**Table 1:** Baseline characteristics of participants stratified by VA groups.

	Overall	SUPERNOVA	Normal VA	EVA	*p*-Value
n	6305	652	5108	545	
Age (years)	62.52 (7.75)	68.94 (8.38)	61.57 (7.28)	63.66 (7.48)	<0.001
Vascular age (years)	61.86 (8.10)	63.23 (7.74)	60.80 (7.48)	70.23 (8.92)	<0.001
Male, n (%)	2156 (34.2)	203 (31.1)	1699 (33.3)	254 (46.6)	<0.001
BMI (kg/m^2^)	25.29 (3.34)	24.81 (3.18)	25.33 (3.36)	25.40 (3.33)	<0.001
SBP (mmHg)	133.18 (16.73)	128.06 (16.95)	132.49 (16.12)	145.76 (16.36)	<0.001
DBP (mmHg)	78.97 (9.60)	73.47 (8.28)	79.28 (9.38)	82.58 (10.53)	<0.001
FBG (mmol/L)	6.21 (1.92)	5.78 (1.23)	6.15 (1.81)	7.26 (3.01)	<0.001
eGFR (ml/min/1.73 m^2^)	93.05 (11.68)	87.59 (11.70)	93.86 (11.39)	92.05 (12.48)	<0.001
TC (mmol/L)	5.33 (1.03)	5.13 (1.01)	5.36 (1.02)	5.32 (1.10)	<0.001
TG (mmol/L)	1.38 (0.98, 1.95)	1.22 (0.89, 1.62)	1.39 (0.99, 1.97)	1.52 (1.05, 2.15)	<0.001
HDL-C (mmol/L)	1.49 (0.35)	1.54 (0.36)	1.49 (0.35)	1.43 (0.34)	<0.001
LDL-C (mmol/L)	3.42 (0.97)	3.24 (0.96)	3.44 (0.97)	3.43 (1.03)	<0.001
Smoking, n (%)					<0.001
No smoking	5017 (80.6)	545 (84.9)	4077 (80.8)	395 (73.4)	
Ever smoking	322 (5.2)	34 (5.3)	252 (5.0)	36 (6.7)	
Current smoking	885 (14.2)	63 (9.8)	715 (14.2)	107 (19.9)	
Drinking, n (%)					<0.001
No drinking	5491 (88.0)	602 (93.8)	4464 (88.2)	425 (78.9)	
Ever drinking	104 (1.7)	9 (1.4)	82 (1.6)	13 (2.4)	
Current drinking	647 (10.4)	31 (4.8)	515 (10.2)	101 (18.7)	
Comorbidities, n (%)					
Hypertension	3476 (55.1)	275 (42.2)	2746 (53.8)	455 (83.5)	<0.001
Dyslipidemia	5082 (80.7)	482 (73.9)	4145 (81.2)	455 (83.5)	<0.001
Diabetes	1872 (29.7)	144 (22.1)	1458 (28.6)	270 (49.5)	<0.001
Myocardial infarction	148 (2.4)	23 (3.6)	99 (2.0)	26 (5.0)	<0.001
Stroke	299 (4.8)	38 (5.8)	219 (4.3)	42 (7.7)	<0.001
Treatment, n (%)					
Lipid-lowering drugs	1276 (20.3)	133 (20.5)	1028 (20.2)	115 (21.2)	0.870
Antihypertensive drugs	2301 (36.6)	194 (29.8)	1807 (35.5)	300 (55.2)	<0.001
Hypoglycemic drugs	1064 (16.9)	90 (13.8)	798 (15.7)	176 (32.4)	<0.001
Target organ damage					
cfPWV (m/s)	8.60 (1.90)	7.11 (1.29)	8.40 (1.37)	12.31 (2.27)	<0.001
Carotid plaque, n (%)	2932 (46.5)	343 (52.6)	2228 (43.6)	361 (66.2)	<0.001
Mean CIMT (mm)	0.73 (0.12)	0.74 (0.12)	0.72 (0.12)	0.77 (0.14)	<0.001
Maximum CIMT (mm)	0.90 (0.15)	0.92 (0.15)	0.89 (0.15)	0.95 (0.17)	<0.001

Notes: Data are presented as mean (SD), median (IQR), or n (%). Abbreviations: cfPWV, carotid-femoral pulse wave velocity; BMI, body mass index; DBP, diastolic blood pressure; SBP, systolic blood pressure; FBG, fasting blood glucose; eGFR, estimated glomerular filtration rate; TC, total cholesterol; TG, triglyceride; HDL-C, high-density lipoprotein cholesterol; LDL-C, low-density lipoprotein cholesterol.

**Table 2:** Multivariable regressions for target organ damage (TOD) according to Δ-age and Δ-age groups.

	Adjust Model 1 β/OR (95%CI)	*p*-Value	Adjust Model 2 β/OR (95%CI)	*p*-Value	Adjust Model 3 β/OR (95%CI)	*p*-Value
Mean CIMT, mm						
Per 1-year increase	0.004 (0.003, 0.005)	<0.001	0.003 (0.002, 0.004)	<0.001	0.006 (0.003, 0.010)	<0.001
SUPERNOVA	−0.016 (−0.025, −0.007)	<0.001	−0.010 (−0.019, −0.000)	0.040	−0.001 (−0.012, 0.010)	0.828
Normal VA	0	Ref.	0	Ref.	0	Ref.
EVA	0.036 (0.026, 0.045)	<0.001	0.030 (0.020, 0.040)	<0.001	0.016 (0.003, 0.029)	0.019
Maximum CIMT, mm						
Per 1-year increase	0.005 (0.004, 0.006)	<0.001	0.004 (0.003, 0.005)	<0.001	0.008 (0.003, 0.012)	<0.001
SUPERNOVA	−0.025 (−0.036, −0.013)	<0.001	−0.016 (−0.027, −0.004)	0.011	−0.005 (−0.019, 0.009)	0.473
Normal VA	0	Ref	0	Ref.	0	Ref.
EVA	0.040 (0.027, 0.052)	<0.001	0.031 (0.019, 0.044)	<0.001	0.015 (−0.002, 0.031)	0.088
Carotid plaque		Ref.				
Per 1-year increase	1.091 (1.072, 1.110)	<0.001	1.065 (1.044, 1.085)	<0.001	1.185 (1.089, 1.290)	<0.001
SUPERNOVA	0.738 (0.611, 0.893)	0.0017	0.815 (0.668, 0.994)	0.044	0.928 (0.736, 1.169)	0.525
Normal VA	1.000	Ref.	1.000	Ref.	1.000	Ref.
EVA	2.111 (1.729, 2.578)	<0.001	1.752 (1.418, 2.165)	<0.001	1.419 (1.070, 1.881)	0.015

Model 1: adjusted for age and sex. Model 2: adjusted for age, sex, body mass index, estimated glomerular filtration rate, smoking and drinking habits, use of antihypertensive drugs, hypoglycemic drugs, lipid-lowering drugs, history of hypertension, diabetes, hyperlipidemia, myocardial infarction, stroke. Model 3 included all components of model 2 plus cfPWV. Abbreviations: VA, vascular aging; SUPERNOVA, supernormal vascular aging; EVA, early vascular aging; CIMT, carotid intima-media thickness; OR, odds ratio; Ref.: Reference group; Δ-age: vascular age minus chronological age. Shortcuts: Δ-age ↑ → mean CIMT ↑ (β per year); max CIMT ↑ (β per year); carotid plaque ↑ (OR per year); EVA > normal VA (CIMT/plaque); SUPERNOVA < normal VA; independent of cfPWV. (Δ-age↑: increase in Δ-age).

## Data Availability

Data cannot be shared publicly, because data from this study may contain potentially or sensitive patient information.

## Supplementary Materials

The following supporting information can be downloaded at: https://www.mdpi.com/article/10.3390/jcdd13010056/s1, Table S1: Distribution of Δ-age and VA categories across 10-year age groups; Table S2: Multivariable regressions for target organ damage (TOD) according to Δ-age and Δ-age groups, additionally adjusted for urine albumin-to-creatinine ratio (UACR); Table S3: Multivariable regressions for target organ damage (TOD) according to Δ-age and Δ-age groups, additionally adjusted for combined CKD staging of eGFR (G stage) and albuminuria (A stage); Table S4: Multivariable regressions for target organ damage (TOD) according to dichotomized Δ-age (≥0 vs. <0); Table S5: Multivariable regressions for target organ damage (TOD) according to Δ-age quartiles; Table S6: The association between Δ-age and target organ damage in subgroups. References [39,40,41] are cited in the Supplementary Materials.

## Abbreviations

The following abbreviations are used in this manuscript:

BMI	body mass index
cfPWV	carotid-femoral pulse wave velocity
CIMT	carotid intima-media thickness
CVD	cardiovascular disease
DBP	diastolic blood pressure
eGFR	estimated glomerular filtration rate
EVA	early vascular aging
FBG	fasting blood glucose
HDL-C	high-density lipoprotein cholesterol
HVA	healthy vascular aging
LDL-C	low-density lipoprotein cholesterol
PWV	pulse wave velocity
SBP	systolic blood pressure
SUPERNOVA	supernormal vascular aging
TC	total cholesterol
TG	triglyceride
TOD	target organ damage
VA	vascular aging

## References

[1] Ahmadi-Abhari S., Sabia S., Shipley M.J., Kivimaki M., Singh-Manoux A., Tabak A., McEniery C., Wilkinson I., Brunner E.J. (2017). Physical Activity, Sedentary Behavior, and Long-Term Changes in Aortic Stiffness: The Whitehall II Study. J. Am. Heart Assoc..

[2] Scuteri A., Morrell C.H., Orrù M., Strait J.B., Tarasov K.V., Ferreli L.A.P., Loi F., Pilia M.G., Delitala A., Spurgeon H. (2014). Longitudinal Perspective on the Conundrum of Central Arterial Stiffness, Blood Pressure, and Aging. Hypertension.

[3] Kaess B.M.R.J., Larson M.G., Hamburg N.M., Vita J.A., Levy D., Benjamin E.J., Vasan R.S., Mitchell G.F. (2012). Aortic stiffness, blood pressure progression, and incident hypertension. JAMA.

[4] Chen Y., Dangardt F., Osika W., Berggren K., Gronowitz E., Friberg P. (2012). Age- and sex-related differences in vascular function and vascular response to mental stress. Longitudinal and cross-sectional studies in a cohort of healthy children and adolescents. Atherosclerosis.

[5] Kucharska-Newton A.M., Stoner L., Meyer M.L. (2019). Determinants of Vascular Age: An Epidemiological Perspective. Clin. Chem..

[6] Wang Y., Wang J., Zheng X.W., Du M.F., Zhang X., Chu C., Wang D., Liao Y.-Y., Ma Q., Jia H. (2023). Early-Life Cardiovascular Risk Factor Trajectories and Vascular Aging in Midlife: A 30-Year Prospective Cohort Study. Hypertension.

[7] Csiszar A.P.A., Wolin M.S., Losonczy G., Pacher P., Ungvari Z. (2009). Oxidative stress and accelerated vascular aging: Implications for cigarette smoking. Front. Biosci..

[8] Zuo Y., Chen S., Tian X., Wang P., Wu S., Wang A. (2023). Association of Vascular Aging with Cardiovascular Disease in Middle-Aged Chinese People: A Prospective Cohort Study. JACC Asia.

[9] Watanabe D., Gando Y., Murakami H., Kawano H., Yamamoto K., Morishita A., Miyatake N., Miyachi M. (2023). Longitudinal trajectory of vascular age indices and cardiovascular risk factors: A repeated-measures analysis. Sci. Rep..

[10] Mitchell G.F., Rong J., Larson M.G., Korzinski T.J., Xanthakis V., Sigurdsson S., Gudnason V., Launer L.J., Aspelund T., Hamburg N.M. (2024). Vascular Age Assessed From an Uncalibrated, Noninvasive Pressure Waveform by Using a Deep Learning Approach: The AI-VascularAge Model. Hypertension.

[11] Ji H., Teliewubai J., Lu Y., Xiong J., Yu S., Chi C., Li J., Blacher J., Zhang Y., Xu Y. (2018). Vascular aging and preclinical target organ damage in community-dwelling elderly: The Northern Shanghai Study. J. Hypertens..

[12] Vasan R.S., Short M.I., Niiranen T.J., Xanthakis V., DeCarli C., Cheng S., Seshadri S., Mitchell G.F. (2019). Interrelations Between Arterial Stiffness, Target Organ Damage, and Cardiovascular Disease Outcomes. J. Am. Heart Assoc..

[13] Fan F., Qi L., Jia J., Xu X., Liu Y., Yang Y., Qin X., Li J., Li H., Zhang Y. (2016). Noninvasive Central Systolic Blood Pressure Is More Strongly Related to Kidney Function Decline Than Peripheral Systolic Blood Pressure in a Chinese Community-Based Population. Hypertension.

[14] Bruno R.M., Nilsson P.M., Engstrom G., Wadstrom B.N., Empana J.P., Boutouyrie P., Laurent S. (2020). Early and Supernormal Vascular Aging: Clinical Characteristics and Association With Incident Cardiovascular Events. Hypertension.

[15] Zanelli S., Agnoletti D., Alastruey J., Allen J., Bianchini E., Bikia V., Boutouyrie P., Bruno R.M., Climie R., Djeldjli D. (2024). Developing technologies to assess vascular ageing: A roadmap from VascAgeNet. Physiol. Meas..

[16] Kim H.L., Lim W.H., Seo J.B., Kim S.H., Zo Z.H., Kim M.A. (2020). Prediction of cardiovascular events using brachial-ankle pulse wave velocity in hypertensive patients. J. Clin. Hypertens..

[17] Sequí-Domínguez I., Cavero-Redondo I., Álvarez-Bueno C., Pozuelo-Carrascosa D.P., Nuñez de Arenas-Arroyo S., Martínez-Vizcaíno V. (2020). Accuracy of Pulse Wave Velocity Predicting Cardiovascular and All-Cause Mortality. A Systematic Review and Meta-Analysis. J. Clin. Med..

[18] Kim H.-L., Lee K.-S., Joh H.S., Lim W.-H., Seo J.-B., Kim S.-H., Zo J.-H., Kim M.-A. (2023). Prognostic Value of Brachial-Ankle Pulse Wave Velocity According to Subjects’ Clinical Characteristics: Data From Analysis of 10,597 Subjects. J. Korean Med. Sci..

[19] Lin C.-C., Li C.-I., Liu C.-S., Lin C.-H., Yang S.-Y., Li T.-C. (2022). Prediction of all-cause and cardiovascular mortality using ankle-brachial index and brachial-ankle pulse wave velocity in patients with type 2 diabetes. Sci. Rep..

[20] Tanaka H., Munakata M., Kawano Y., Ohishi M., Shoji T., Sugawara J., Tomiyama H., Yamashina A., Yasuda H., Sawayama T. (2009). Comparison between carotid-femoral and brachial-ankle pulse wave velocity as measures of arterial stiffness. J. Hypertens..

[21] Reference Values for Arterial Stiffness’ Collaboration (2010). Determinants of pulse wave velocity in healthy people and in the presence of cardiovascular risk factors: ‘Establishing normal and reference values’. Eur. Heart J..

[22] Williams B., Mancia G., Spiering W., Agabiti Rosei E., Azizi M., Burnier M., Clement D.L., Coca A., de Simone G., Dominiczak A. (2018). 2018 ESC/ESH Guidelines for the management of arterial hypertension. Eur. Heart J..

[23] Mancia G.K.R., Brunström M., Burnier M., Grassi G., Januszewicz A., Muiesan M.L., Tsioufis K., Agabiti-Rosei E., Algharably E.A.E., Azizi M. (2023). 2023 ESH Guidelines for the management of arterial hypertension The Task Force for the management of arterial hypertension of the European Society of Hypertension: Endorsed by the International Society of Hypertension (ISH) and the European Renal Association (ERA). J. Hypertens..

[24] Mitchell G.F., Hwang S.J., Vasan R.S., Larson M.G., Pencina M.J., Hamburg N.M., Vita J.A., Levy D., Benjamin E.J. (2010). Arterial stiffness and cardiovascular events: The Framingham Heart Study. Circulation.

[25] Ng X.-N.T.J.-P., Wang C.-H., Hsu B.-G. (2023). Carotid–Femoral Pulse Wave Velocity Could Be a Marker to Predict Cardiovascular and All-Cause Mortality of Hemodialysis Patients. J. Clin. Med..

[26] Tougaard N.H., Theilade S., Winther S.A., Tofte N., Ahluwalia T.S., Hansen T.W., Rossing P., Frimodt-Møller M. (2020). Carotid-Femoral Pulse Wave Velocity as a Risk Marker for Development of Complications in Type 1 Diabetes Mellitus. J. Am. Heart Assoc..

[27] Tan J., Pei Y., Hua Q., Xing X., Wen J. (2014). Aortic pulse wave velocity is associated with measures of subclinical target organ damage in patients with mild hypertension. Cell Biochem. Biophys..

[28] Bai Y., Wang Q., Cheng D., Hu Y., Chao H., Avolio A., Tang B., Zuo J. (2022). Comparison of Risk of Target Organ Damage in Different Phenotypes of Arterial Stiffness and Central Aortic Blood Pressure. Front. Cardiovasc. Med..

[29] Mantella L.E., Colledanchise K.N., Hetu M.F., Feinstein S.B., Abunassar J., Johri A.M. (2019). Carotid intraplaque neovascularization predicts coronary artery disease and cardiovascular events. Eur. Heart J. Cardiovasc. Imaging.

[30] Mantella L.E., Colledanchise K.N., Wheatley M.G.A., McCreath P., Suri J.S., Hetu M.F., Johri A.M. (2023). A Novel Ultrasound-Based Carotid Plaque Vulnerability Score Is Associated with Long-Term Cardiovascular Outcomes. J. Am. Soc. Echocardiogr..

[31] van Oostrom O., Velema E., Schoneveld A.H., de Vries J.P., de Bruin P., Seldenrijk C.A., de Kleijn D.P., Busser E., Moll F.L., Verheijen J.H. (2005). Age-related changes in plaque composition: A study in patients suffering from carotid artery stenosis. Cardiovasc. Pathol..

[32] Park S.J., Kang S.J., Ahn J.M., Chang M., Yun S.C., Roh J.H., Lee P.H., Park H.W., Yoon S.H., Park D.W. (2016). Effect of Statin Treatment on Modifying Plaque Composition: A Double-Blind, Randomized Study. J. Am. Coll. Cardiol..

[33] Lima J.A., Desai M.Y., Steen H., Warren W.P., Gautam S., Lai S. (2004). Statin-induced cholesterol lowering and plaque regression after 6 months of magnetic resonance imaging-monitored therapy. Circulation.

[34] Hattori K., Ozaki Y., Ismail T.F., Okumura M., Naruse H., Kan S., Ishikawa M., Kawai T., Ohta M., Kawai H. (2012). Impact of Statin Therapy on Plaque Characteristics as Assessed by Serial OCT, Grayscale and Integrated Backscatter–IVUS. JACC Cardiovasc. Imaging.

[35] Formanowicz D., Krawczyk J.B., Perek B., Lipski D., Tykarski A. (2021). Management of High-Risk Atherosclerotic Patients by Statins May Be Supported by Logistic Model of Intima-Media Thickening. J. Clin. Med..

[36] Dudenbostel T., Glasser S.P. (2012). Effects of antihypertensive drugs on arterial stiffness. Cardiol. Rev..

[37] Kim H., Choi C.U., Rhew K., Park J., Lim Y., Kim M.G., Kim K. (2024). Comparative effects of glucose-lowering agents on endothelial function and arterial stiffness in patients with type 2 diabetes: A network meta-analysis. Atherosclerosis.

[38] Izzo C., Vitillo P., Di Pietro P., Visco V., Strianese A., Virtuoso N., Ciccarelli M., Galasso G., Carrizzo A., Vecchione C. (2021). The Role of Oxidative Stress in Cardiovascular Aging and Cardiovascular Diseases. Life.

[39] Ogihara T.K.K., Matsuoka H., Fujita T., Higaki J., Horiuchi M., Imai Y., Imaizumi T., Ito S., Iwao H., Kario K. (2009). The Japanese Society of Hypertension Guidelines for the Management of Hypertension (JSH 2009). Hypertens. Res..

[40] Alberti K.G.Z.P. (1998). Definition, diagnosis and classification of diabetes mellitus and its complications. Part 1: Diagnosis and classification of diabetes mellitus provisional report of a WHO consultation. Diabet. Med..

[41] Li J.J., Zhao S.P., Zhao D., Lu G.P., Peng D.Q., Liu J., Chen Z.-Y., Guo Y.-L., Wu N.-Q., Yan S.-K. (2023). 2023 Chinese guideline for lipid management. Front Pharmacol..

