# Supplementary material for: Association of Vascular Age and Subclinical Target Organ Damage in a Beijing Community-Based Population: A Cross-Sectional Study
Source: J Cardiovasc Dev Dis. 2026 Jan 21;13(1):56. doi: 10.3390/jcdd13010056 (PMC12842185; doi:10.3390/jcdd13010056)
Supplement: Supplementary file 1 [file jcdd-13-00056-s001.zip › jcdd-4007531-supplementary.pdf]

Supplementary Material

Association of Vascular Age and Subclinical Target Organ Damage in a Beijing Community-based Population: A Cross-sectional Study

Supplementary Methods. Additional details regarding methods 2

Table S1. Distribution of Δ-age and VA categories across 10-year age groups 3

Table S2. Multivariable regressions for target organ damage (TOD) according to Δ-age and Δ-age groups, additionally adjusted for urine albumin-to-creatinine ratio (UACR) 4

Table S3. Multivariable regressions for target organ damage (TOD) according to Δ-age and Δ-age groups, additionally adjusted for combined CKD staging of eGFR (G stage) and albuminuria (A stage) 6

Table S4. Multivariable regressions for target organ damage (TOD) according to dichotomized Δ-age (≥0 vs. <0) 8

Table S5. Multivariable regressions for target organ damage (TOD) according to Δ-age quartiles 9

Table S6. The association between Δ-age and target organ damage in subgroups 10

Supplementary References 14

Supplementary Methods. Additional details regarding methods.

Hypertension was defined as a self-reported history of hypertension, current use of antihypertensive drugs, or a measured average systolic blood pressure (SBP) ≥140 mmHg and/or diastolic blood pressure (DBP) ≥90 mmHg (average of three readings, at least 1 minute apart)(1). Diabetes was defined as self-reported diabetes, current hypoglycemic therapy, fasting blood glucose ≥7.0 mmol/L, or 2-hour postprandial glucose ≥11.1 mmol/L(2). Hyperlipidemia was defined as self-reported hyperlipidemia, current lipid-lowering therapy, or abnormal lipid levels (total cholesterol [TC] ≥5.18 mmol/L, triglyceride [TG] ≥1.70 mmol/L, low-density lipoprotein cholesterol [LDL-C] ≥3.37 mmol/L, or high-density lipoprotein cholesterol [HDL-C] <1.04 mmol/L). CVD was defined as a self-reported history of myocardial infarction and/or stroke(3).

Table S1. Distribution of Δ-age and VA categories across 10-year age groups.

Age group (years)	⩽40	>40, ⩽50	>50, ⩽60	>60, ⩽70	>70, ⩽80	>80	P-value
**n**	17	251	2354	2780	753	150	
**Δ-age (years)**	-1.000 (-2.000, -0.000)	-1.000 (-2.000, -0.500)	-0.500 (-2.000, -1.000)	-1.000 (-3.000, -1.000)	-2.000 (-4.500, -0.667)	-3.000 (-6.500, -0.375)	<0.001
**Δ-age categories**							<0.001
SUPERNOVA, n(%)	16 (94.1)	234 (93.2)	2085 (88.6)	2220 (79.9)	479 (63.6)	74 (49.3)	
Normal VA, n(%)	1 (5.9)	5 (2.0)	94 (4.0)	289 (10.4)	203 (27.0)	60 (40.0)	
EVA, n(%)	0 (0)	12 (4.8)	175 (7.4)	271 (9.7(	71 (9.4)	16 (10.7)	

Notes: Data are presented as median (IQR) or n (%).

Abbreviations: VA, vascular aging; SUPERNOVA: supernormal vascular aging; EVA: early vascular aging; IQR: interquartile range.

Table S2. Multivariable regressions for target organ damage (TOD) according to Δ-age and Δ-age groups, additionally adjusted for urine albumin-to-creatinine ratio (UACR).

	Adjust model β/OR(95%CI)	P-value
**Mean CIMT, mm**		
**Per 1-year increase**	0.006 (0.003, 0.010)	<0.001
**SUPERNOVA**	-0.001 (-0.012, 0.010)	0.839
**Normal VA**	0	
**EVA**	0.016 (0.003, 0.029)	0.020
**Maximum CIMT, mm**		
**Per 1-year increase**	0.008 (0.003, 0.012)	<0.001
**SUPERNOVA**	-0.005 (-0.019, 0.009)	0.481
**Normal VA**	0	
**EVA**	0.015 (-0.002, 0.031)	0.090
**Carotid plaque**		
**Per 1-year increase**	1.185 (1.089, 1.290)	<0.001
**SUPERNOVA**	0.928 (0.736, 1.169)	0.512
**Normal VA**	1.000	

**EVA**	1.419 (1.070, 1.881)	0.015

* Adjusted for age, sex, body mass index, estimated glomerular filtration rate, smoking and drinking habits, use of antihypertensive drugs, hypoglycemic drugs, lipid-lowering drugs, history of hypertension, diabetes, hyperlipidemia, myocardial infarction, stroke, cfPWV, and UACR. Abbreviations: VA, vascular age; SUPERNOVA, supernormal vascular aging; EVA, early vascular aging; CIMT, carotid intima-media thickness; OR, odds ratio.

Table S3. Multivariable regressions for target organ damage (TOD) according to Δ-age and Δ-age groups, additionally adjusted for combined CKD staging of eGFR (G stage) and albuminuria (A stage)

	Adjust model β/OR(95%CI)	P-value
**Mean CIMT, mm**		
**Per 1-year increase**	0.006 (0.003, 0.010)	<0.001
**SUPERNOVA**	-0.002 (-0.013, 0.009)	0.757
**Normal VA**	0	
**EVA**	0.016 (0.003, 0.029)	0.019
**Maximum CIMT, mm**		
**Per 1-year increase**	0.008 (0.003, 0.012)	<0.001
**SUPERNOVA**	-0.006 (-0.019, 0.008)	0.424
**Normal VA**	0	
**EVA**	0.015 (-0.002, 0.031)	0.090
**Carotid plaque**		
**Per 1-year increase**	1.179 (1.081, 1.285)	<0.001
**SUPERNOVA**	0.946 (0.750, 1.193)	0.638
**Normal VA**	1.000	

**EVA**	1.420 (1.070, 1.884)	0.015

* Adjusted for age, sex, body mass index, smoking and drinking habits, use of antihypertensive drugs, hypoglycemic drugs, lipid-lowering drugs, history of hypertension, diabetes, hyperlipidemia, myocardial infarction, stroke, cfPWV, and CKD staging.

Abbreviations: VA, vascular age; SUPERNOVA, supernormal vascular aging; EVA, early vascular aging; CIMT, carotid intima-media thickness; OR, odds ratio

Table S4. Multivariable regressions for target organ damage (TOD) according to dichotomized Δ-age (≥0 vs. <0)

		Mean CIMT		Maximum CIMT		Carotid plaque	
	N	Adjust model β/OR (95%CI)	P value	Adjust model β/OR (95%CI)	P value	Adjust model β/OR (95%CI)	P value
**Δ-age category (years)**							
<0	3767	0	Ref.	0	Ref.	1.000	Ref.
≥0	2538	0.004 (-0.004, 0.012)	0.338	0.005 (-0.006, 0.015)	0.372	1.093 (0.917, 1.304)	0.321

*Adjusted for age, sex, body mass index, estimated glomerular filtration rate, smoking and drinking habits, use of antihypertensive drugs, hypoglycemic drugs, lipid-lowering drugs, history of hypertension, diabetes, hyperlipidemia, myocardial infarction, stroke, and carotid-femoral pulse wave velocity.

Abbreviations: CIMT, carotid intima-media thickness; OR, odds ratio.

Table S5. Multivariable regressions for target organ damage (TOD) according to Δ-age quartiles

	N	Mean CIMT		Maximum CIMT		Carotid plaque	
		Adjust model β/OR (95%CI)	P value	Adjust model β/OR (95%CI)	P value	Adjust model β/OR (95%CI)	P value
**Δ-age quartile**							
Q1 (≤-3.00 years)	1203	0	Ref.	0	Ref.	1.000	Ref.
Q2 (>-3.00 to ≤-1.00 years)	1711	0.003 (-0.006, 0.013)	0.450	0.009 (-0.003, 0.020)	0.143	1.003 (0.827, 1.218)	0.972
Q3 (>-1.00 to ≤ 1.00 years)	1649	0.003 (-0.008, 0.013)	0.631	0.009 (-0.004, 0.023)	0.188	1.240 (0.980, 1.570)	0.073
Q4 (>1.00 years)	1742	0.015 (-0.000, 0.029	0.051	0.021 (0.003, 0.040)	0.026	1.288 (0.928, 1.789)	0.131

*Adjusted for age, sex, body mass index, estimated glomerular filtration rate, smoking and drinking habits, use of antihypertensive drugs, hypoglycemic drugs, lipid-lowering drugs, history of hypertension, diabetes, hyperlipidemia, myocardial infarction, stroke, and carotid-femoral pulse wave velocity.

Abbreviations: CIMT, carotid intima-media thickness; OR, odds ratio.

Table S6. The association between Δ-age and target organ damage in subgroups.

		Mean CIMT			Maximum CIMT			Carotid plaque		
	N	Adjust model β/OR (95%CI)	P value	P for interaction	Adjust model β/OR (95%CI)	P value	P for interaction	Adjust model β/OR (95%CI)	P value	P for interaction
**Age category (years)**				0.203			0.613			0.270
<60	2166	0.006 (0.001, 0.010)	0.009		0.006 (0.001, 0.012)	0.020		1.232 (1.109, 1.369)	<0.001	
≥60	3868	0.004 (0.000, 0.008)	0.032		0.006 (0.001, 0.011)	0.026		1.202 (1.09, 1.326)	<0.001	
**Sex**				0.689			0.316			0.941
Male	2052	0.006 (0.002, 0.010)	0.001		0.007 (0.002, 0.012)	0.003		1.184 (1.084, 1.293)	<0.001	
Female	3982	0.006 (0.003, 0.010)	0.001		0.008 (0.003, 0.013)	0.001		1.186 (1.088, 1.292)	<0.001	
**BMI categorical (kg/m^2)**				0.935			0.699			0.506

<24	2204	0.006 (0.003, 0.010)	<0.001		0.008 (0.003, 0.013)	<0.001		1.173 (1.075, 1.280)	<0.001	
≥24, <28	2671	0.006 (0.002, 0.010)	0.001		0.007 (0.003, 0.012)	0.002		1.199 (1.098, 1.309)	<0.001	
≥28	1159	0.006 (0.002, 0.010)	0.004		0.007 (0.002, 0.012)	0.007		1.175 (1.071, 1.290)	<0.001	
**EGFR category (ml/min/1.73 m^2)**				0.651			1.000			0.994
<90	1796	0.006 (0.003, 0.010)	<0.001		0.008 (0.003, 0.012)	<0.001		1.185 (1.087, 1.292)	<0.001	
>=90	4238	0.007 (0.003, 0.010)	<0.001		0.008 (0.003, 0.012)	0.001		1.185 (1.083, 1.296)	<0.001	
**Smoking**				0.727			0.666			0.338
No smoking	4866	0.006 (0.003, 0.010)	<0.001		0.008 (0.003, 0.012)	<0.001		1.185 (1.089, 1.291)	<0.001	
Ever smoking	311	0.006 (0.002, 0.011)	0.009		0.008 (0.002, 0.014)	0.010		1.23 (1.099, 1.376)	<0.001	
Current smoking	857	0.005 (0.001, 0.009)	0.008		0.007 (0.002, 0.012)	0.011		1.154 (1.049, 1.270)	0.003	

**Drinking**				0.671			0.599			0.206
No drinking	5314	0.006 (0.003, 0.010)	<0.001		0.008 (0.003, 0.012)	<0.001		1.180 (1.084, 1.285)	<0.001	
Ever drinking	98	0.004 (-0.003, 0.010)	0.260		0.004 (-0.004, 0.012)	0.327		1.311 (1.105, 1.556)	0.002	
Current drinking	622	0.006 (0.002, 0.010)	0.003		0.008 (0.002, 0.013)	0.004		1.218 (1.102, 1.346)	<0.001	
**History of hypertension**				0.083			0.116			0.144
No	2733	0.007 (0.004, 0.011)	<0.001		0.009 (0.004, 0.014)	<0.001		1.209 (1.106, 1.322)	<0.001	
Yes	3301	0.006 (0.002, 0.009)	0.002		0.007 (0.003, 0.012)	0.002		1.175 (1.078, 1.280)	<0.001	
**History of dyslipidemia**				0.464			0.610			0.169
No	1136	0.007 (0.003, 0.011)	<0.001		0.008 (0.003, 0.013)	0.001		1.157 (1.055, 1.268)	0.002	
Yes	4898	0.006 (0.002, 0.010)	0.001		0.007 (0.003, 0.012)	0.001		1.193 (1.095, 1.299)	<0.001	

**History of diabetes**				0.635			0.933			0.860
No	4272	0.006 (0.003, 0.01)	<0.001		0.008 (0.003, 0.012)	0.001		1.184 (1.086, 1.29)	<0.001	
Yes	1762	0.006 (0.002, 0.01)	0.002		0.008 (0.003, 0.012)	0.005		1.187 (1.087, 1.297)	<0.001	
**History of CVD**				0.273			0.263			0.400
No	5634	0.006 (0.002, 0.010)	0.001		0.007 (0.003, 0.012)	0.001		1.180 (1.084, 1.285)	<0.001	
Yes	400	0.007 (0.003, 0.012)	<0.001		0.009 (0.004, 0.014)	<0.001		1.214 (1.095, 1.345)	<0.001	

*Adjusted for age, sex, body mass index, estimated glomerular filtration rate, smoking and drinking habits, use of antihypertensive drugs, hypoglycemic drugs, lipid-lowering drugs, history of hypertension, diabetes, hyperlipidemia, myocardial infarction, stroke, and carotid-femoral pulse wave velocity.

Abbreviations: CIMT, carotid intima-media thickness; EGFR, estimated glomerular filtration rate; BMI, body mass index; CVD, cardiovascular disease; OR, odds ratio.

Supplementary References

1. Ogihara T KK, Matsuoka H, Fujita T, Higaki J, Horiuchi M, et al. The Japanese Society of Hypertension Guidelines for the Management of Hypertension (JSH 2009). Hypertens Res. 2009;32:3-107.
2. Alberti KG ZP. Definition, diagnosis and classification of diabetes mellitus and its complications. Part 1: diagnosis and classification of diabetes mellitus provisional report of a WHO consultation. Diabet Med. 1998;15:539-53.
3. Li JJ, Zhao SP, Zhao D, Lu GP, Peng DQ, Liu J, et al. 2023 Chinese guideline for lipid management. Front Pharmacol. 2023;14:1190934.
